# Clinical benefits of early-stage autologous conditioned serum and injectable platelet-rich fibrin on healing superficial digital flexor tendonitis in donkeys

**DOI:** 10.1186/s13620-025-00299-y

**Published:** 2025-06-07

**Authors:** Mahmoud Najeb, Alaa Samy, Awad Rizk, Esam Mosbah, Gamal Karrouf

**Affiliations:** Department of Surgery, Anesthesiology, and Radiology, Faculty of Veterinary Medicine, Mansoura University, Mansoura, 35516 Egypt

**Keywords:** Injectable platelet-rich fibrin, Autologous conditioned serum, Superficial digital flexor tendon, Interleukin-1 receptor antagonist protein

## Abstract

Injectable platelet-rich fibrin (I-PRF) has been explored for its regenerative potential, but its application in tendon healing remains unstudied. Autologous conditioned serum (ACS), commonly used in joint therapies, inhibits interleukin 1-mediated inflammation, though its role in tendon healing is not well established. This study aimed to evaluate the clinical benefits of I-PRF alone and in combination with ACS in tendon healing. Twenty-three donkeys with forelimb superficial digital flexor (SDF) tendonitis were examined at admission (T0) and randomly assigned to PRF/ACS treated group (*n* = 10), PRF group (*n* = 7), or included as a control group (*n* = 6). Among clinical parameters, tendon shape and weight-bearing scores at T150 were significantly improved (*P* < 0.001) in the PRF/ACS group compared to the control group. Regardless of the tendon cross-sectional area (T-CSA), all other ultrasonographic parameters showed significant improvement in the PRF/ACS group compared to the control group. T-CSA increased throughout the study in PRF group but significantly decreased at T150 in control and PRF/ACS groups. The combination therapy had a significant improvement in fiber alignment score (FAS) and T-CSA compared to PRF group. Early lesion resolution in PRF/ACS at T90 and later in PRF at T150 suggests superior healing in PRF/ACS group. Persistent increase in T-CSA in the PRF group indicates ongoing healing, while its reduction in the PRF/ACS groups reflects more progressed healing stages and superior outcomes. Conversely, the presence of hyperechoic dots and misaligned tendon fibers in the control group suggests inferior healing outcomes. Intralesional I-PRF combined with ACS significantly enhanced clinical and ultrasonographic healing characteristics in donkeys with SDF tendonitis.

**Supplementary Information:**

The online version contains supplementary material available at 10.1186/s13620-025-00299-y.

## Introduction

Tendon injuries remain a major concern in equine and are often career-ending. The prevalence of SDF tendonitis is particularly high among performance and racehorses, with reported incidence rates ranging from 11 to 43% [1]. These injuries are challenging to treat, as tendons heal through fibrotic scar rather than true regeneration. Once severely damaged, they rarely regain their original elasticity and functional capacity, leading to increasing reinjury rates of up to 80% with conservative treatment [2, 3].

Regenerative therapies, such as platelet concentrates and stem cell therapy, present promising alternatives to traditional treatments for SDF tendonitis [4]. Platelet concentrates are more widely used for managing tendon disorders [5]. Among platelet concentrates, platelet-rich plasma (PRP) has shown beneficial effects in experimental models [6, 7]. However, its efficacy in equine tendon healing remains debated. A systemic review involved eight studies reported that PRP improved structural integrity, reduced lameness, enhanced tissue organization, and led to better performance outcomes [8]. In contrast, a recent meta-analysis of fifteen studies found no definitive evidence that PRP significantly enhances tendon healing in horses [9].

Platelet-rich fibrin (PRF), a second-generation platelet derivative, offers several advantages over PRP. It can be easily prepared stall-side from autologous blood without the need for complex equipment or prolonged processing. PRF contains higher levels of various growth factors with a sustained release of up to 14 days, compared to just four days in PRP [10, 11]. Additionally, its fibrin matrix supports cellular migration, and it is free from chemicals that could interfere with therapeutic activity [12]. While PRF has not been specifically evaluated for treating equine SDF tendonitis, studies have reported varied outcomes in its use for treatment of other tendinopathies [13–16]. Injectable PRF (I-PRF) demonstrated promising regenerative potential in human dentistry [17]. To the authors’ knowledge, this study is the first to assess the clinical application of I-PRF for the treatment of equine SDF tendonitis.

Despite their regenerative potential in tendon healing, their efficacy in equine tendons remains uncertain [9]. This inconsistency may be attributed to excessive and uncontrolled inflammation in equine tendons, which could hinder the expected regenerative effects of platelet-rich derivatives [18]. Effective inflammation control is essential for promoting tendon regeneration and minimizing excessive scar formation [19]. The early tendon repair phase is marked by inflammation and tissue proliferation, which, if uncontrolled, can accelerate tendon fiber degradation. Simultaneously, dysregulated collagen synthesis favors the deposition of weaker type III collagen over type I, leading to disorganized scar formation [20].

Autologous conditioned serum (ACS) is a promising treatment for tendon healing which are crucial for counteracting inflammation and promoting tissue repair, it enhances the concentration of key cytokines and growth factors compared to regular blood, with interleukin 1 receptor antagonist (IL-1Ra) being a significant component [21–23]. ACS specifically inhibits IL-1-mediated inflammation, reducing matrix degradation and pain, and preserving tissue integrity [24]. Although ACS products are commonly used in joint therapies, their application in tendon injuries remains debated among equine clinicians [5]. Horses with SDF tendinopathy treated with ACS showed increased type I collagen content compared to saline-treated tendons, suggesting a potential improvement in tissue quality and mechanical properties [25].

Since tendonitis involves both inflammation and tissue degeneration, a combined intralesional specific anti-inflammatory and regenerative approach may yield superior therapeutic outcomes. This study aims to evaluate the therapeutic efficacy of PRF, both alone and in combination with ACS, in SDF tendonitis. The authors hypothesize that ACS treatment will help regulate excessive inflammation, creating a more favorable environment for PRF mediated regeneration potential.

## Materials and methods

### Animals

The inclusion criteria for this study included adult donkeys with a history of unilateral forelimb SDF tendonitis with intact skin. Cases were eligible if clinical signs of inflammation had been reported 6–14 days before presentation. Enrollment was further restricted to donkeys whose owners consented to the study design and who had not received systemic medical treatment for this specific SDFT injury, nor intralesional tendon injections at any point in their lives. The study was conducted between 2021 and 2024, including a total of 23 donkeys (Equus asinus). The study was approved by the Medical Research Ethics Committee, Faculty of Veterinary Medicine, Mansoura University (Approval Code: MU.ACUC. Ph.D.25.04.54). Affected limbs were randomly assigned to one of three groups: the control group (*n* = 6), the PRF-treated group (*n* = 7), or the PRF/ACS-treated group (*n* = 10). Animals underwent a thorough clinical and ultrasonographical evaluations on the admission day (T0) and on 7, 14, 30, 60, 90, and 150 days after treatment.

### Clinical examination

A comprehensive clinical evaluation was semi quantitatively scored to assess lameness, pain response to pressure, and localized heat upon palpation (Table 1). Tendon shape and intensifying weight-bearing ability under static condition were assessed at T0 and T150. Tendon shape upon palpation was graded as follows: 0 for smooth, uniform tendons with normal thickness; 1 for minimal irregularity and slight thickening; 2 for notable irregularity and thickening; and 3 for hard nodular areas. Intensifying weight-bearing ability under static conditions was evaluated by applying a load equivalent to 30% of the animal’s body weight, with scores ranging from 0 for even weight distribution; 1 for slight preference for the opposite limb; 2 for Notable reduction in weight-bearing, notable preference for the opposite limb; and 3 for notable offloading of the affected limb. To ensure objectivity, all clinical evaluations were conducted blindly by two independent observers, and the average scores used for analysis.

**Table 1:** Clinical assessment scores for tendon healing modified according to Carlier et al. and Reix [26, 27]

Score	Description
Heat	
0	Normal findings
1	Mild increase
2	Moderate increase
3	Severe increase
Pain	
0	Normal findings
1	Mild reaction
2	Moderate reaction
3	Severe reaction
Modified clinical score assessment for lameness	
0	Normal gait
1	Mild, intermittent lameness or difficult to observe, regardless of surface
2	Mild lameness, intermittent at a walk but consistently present under certain conditions (e.g., hard surface, weight-bearing)
3	Mildly abnormal gait and/or stiff walk
4	Reluctance to move when motivated/sever lameness at a walk
5	No movement or lying down; limb barely touches the ground (minimal weight-bearing or resting limb in flexion posture)
Tendon shape upon palpation	
0	Smooth uniform normal thickening
1	Minimal irregularity or slight thickening
2	Notable irregularity
3	severe irregularity with a soft consistency
4	Hard nodular area
Intensifying weight bearing (static examination)	
0	Even weight distribution
1	Slight reduction in weight-bearing, slight preference for the opposite limb
2	Notable reduction in weight-bearing, notable preference for the opposite limb
3	Notable offloading of the limb

### Ultrasonographic evaluation

On the scheduled times, all tendons underwent B-mode ultrasonographic examination using a 10 MHz linear transducer (CHISON Digital Ultrasound, iVis 60 EXPERT VET; CHISON Medical imaging Co., Ltd, China) in both transverse and longitudinal orientations. Images were digitally stored and analyzed to assess the degree and progression of structural changes over time. Both fiber echogenicity (FES) and fiber alignment scores (FAS) were evaluated using a scoring method previously described by Carlier et al. [26].

Both tendon cross sectional area (T-CSA) and lesion cross-sectional area (L-CSA) at the maximum injured zone were quantitatively measured (mm^2^) on transverse ultrasound images. Ultrasonographic examination was performed by MN, while the analysis of ultrasonograms was conducted by another examiner (A.S.), who was blinded to the individual treatment modality. Measurements were made three times and the mean was used for statistical analysis. The percentage of the tendon lesion (Lesion %) was calculated by the following equation [lesion % = (L-CSA/T-CSA) × 100]. To address the statistically significant differences in lesion percentage and T-CSA at baseline (T0), proportional changes in T-CSA (P.T-CSA) and lesion percentage (PL%) over time were calculated by standardizing the T0 value to 1 within each group (by dividing T0 by itself), and then dividing each subsequent time point by the corresponding T0 value [28].

### Autogenous injectable PRF (I-PRF) preparation

Briefly, 4 ml of whole blood was collected in a sterile plastic plain tube and immediately centrifuged at 700 rpm for 3 min (RCF = 22×g) at a 45° rotator angle with radius of 40 mm, separating it into an upper I-PRF layer and a lower RBC layer [29]. The I-PRF was injected within 5 min before clotting.

### Autologous conditioned serum (ACS) preparation

Under aseptic conditions, 10 ml of autologous blood was collected into an orthokine^®^vet irap 10 ml syringe system (Orthogen, Düsseldorf, Germany) and incubated at 37 °C for 6 h. It was then centrifuged at 4000 rpm for 10 min (RCF = 716×g), separating into an upper yellowish conditioned serum (ACS) layer and a lower RBC layer. The conditioned serum was aspirated and filtered through a 0.22 μm syringe-driven filter before use [25].

### Therapy

Donkeys were sedated intravenously with acepromazine (0.05 mg/kg, Castran, 15 mg/mL) and xylazine HCl (1.1 mg/kg, Xyla-Ject, 20 mg/mL), administered 20 min apart, followed by butorphanol (0.05 mg/kg, Torbugesic 10 mg/ml). The medial and lateral palmar nerves were anesthetized 2 cm distal to the carpal joints using 2 ml of 2% lidocaine after skin preparation. Tendons were treated under aseptic conditions according to their respective groups, with a fixed 3 ml inoculum administered in all groups. This volume consisted of normal saline in the control group, 1.5 ml of I-PRF and 1.5 ml of saline in the PRF group, and a combination of 1.5 ml I-PRF and 1.5 ml ACS in the PRF/ACS group. Intratendinous injections were performed using 21-gauge needles under ultrasound guidance, inserted laterally and perpendicular to the tendon’s long axis. The inoculum was distributed at the site of maximal lesion size, as well as 0.5 cm proximal and 0.5 cm distal to that point. All groups received a systemic Flunixin meglumine (Flamicure 5%, Pharma Swede, Egypt) at a dose rate of 1.1 mg/kg for 5 successive days.

### Follow-up and controlled exercise

After treatment, a pressure bandage was applied to the metacarpus for 24 h, consisting of a sterile non-adherent gauze layer, followed by cotton padding, and secured with a cohesive elastic wrap. All animals followed a gradually increasing exercise program as described by Bosch et al. [30]. Briefly, following a 6-day stall rest, the exercise program commenced one week post-treatment, starting with a 10-minute walk, with the duration increasing by 10 min every 3 weeks until the study’s conclusion.

### Statistical analysis

Data were analyzed using the Statistical Package for Social Science IBM SPSS software package (Armonk, NY: IBM Corp), version 28.0. The normality of the quantitative variables was determined using the Shapiro–Wilk test. Normally distributed variables, including age, body weight (BW), L-CSA, and T-CSA, were expressed as mean ± SD and assessed for normality using probability plots. A pairwise comparison of quantitative variables between different groups at different times was carried out using two way ANOVA and the subsequent Tukey’s post hoc correction. To compare the T-CSA of the affected limb at T150 with that of the contralateral limb within the same group, an unpaired Student’s t-test was used, whereas comparisons with that of T0 values of the affected limb were performed using a paired Student’s t-test. To enable pairwise comparisons among lesion percentage and T-CSA, the T0 value (the value before treatment) was standardized to 1 within each group by dividing it by itself. Subsequently, changes in lesion percentage and T-CSA over time were calculated by dividing each value at later time points by the corresponding T0 value. Non-parametric data (pain, heat, lameness, tendon shape, and intensifying weight-carrying scores, FES, FAS) were expressed as median (minimum–maximum). A pairwise comparison of these non-parametric variables between different groups at the same time was carried out using the Kruskal-Wallis non-parametric ANOVA. Concerning pain, heat, lameness, FES, and FAS the Friedman multiple comparison test was used to analyze the effects of different times within the same group, while the Wilcoxon test was used to identify the statistical difference between T0 and T150 among the tendon shape and intensifying weight-carrying scores within the same group. Results were considered statistically significant at *P* < 0.05. All graphs were performed using Graph Pad Prism version (8.4.3., Software Inc., La Jolla, CA).

## Results

### Description and history of donkeys

A total of 23 donkeys (Equus asinus), aged between 3 and 7 years (4.91 ± 1.31) and weighted 185.78 ± 12.91 kg, met the inclusion criteria and were randomly assigned to one of three treatment groups: the control group (*n* = 6), the PRF group (*n* = 7), and the PRF/ACS group (*n* = 10). The underlying cause of tendonitis was primarily attributed to strain due to heavy-drafting activity, affecting 18 of the 23 donkeys (78.3%). This was observed in 8 of 10 SDFTs (80%) in the PRF/ACS group, 5 of 7 SDFTs (71.4%) in the PRF group, and 5 of 6 SDFTs (83.3%) in the control group. Traumatic-induced tendonitis was historically diagnosed in five cases (21.7%), with one case assigned to the control group and two cases in each of the PRF and PRF/ACS groups (Table 2).

**Table 2:** Description, clinical history, and diagnostic data of 23 donkeys with SDFT lesions

	Groups			
	Total	Control	PRF	PRF/ACS
Count	23	6	7	10
Age	4.91 ± 1.31	4.50 ± 1.05	4.86 ± 1.57	5.20 ± 1.32
Body weight (Kg)	185.78 ± 12.91	187.19 ± 9.51	176.742 ± 8.09	184.28 ± 13.83
Gender	Jk (*n* = 18)	5	5	8
	G (*n* = 3)	1	2	1
	Jn (*n* = 1)	--	--	1
Affected limb	RF (*n* = 9)	2	3	4
	LF (*n* = 14)	4	4	6
Cause	St (*n* = 18)	5	5	8
	Tr (*n* = 5)	1	2	2
Lesion type	Core (*n* = 14)	4	4	6
	Marginal (*n* = 9)	2	3	4
Maximal injury zone	1B (*n* = 3)	1	--	2
	2 A (*n* = 9)	2	3	4
	2B (*n* = 11)	3	4	4
Duration of tendonitis until initial admission (days)	8.22 ± 2.75	7.8 ± 3.12	8.14 ± 2.73	8.5 ± 2.8

*Jk* Jackass, *G* Gelding, *Jn* Jennet, *RF* right-Fore limb, *LF* left-Fore limb, *St* strain induced, *Tr* traumatic induced

### Clinical findings

On the day of admission (T0), pain and heat scores across all groups ranged from mild to normal [Score (S) = 0 (0–1)], with most animals (*n* = 16/23) within the normal range and a few (*n* = 7/23) exhibiting mild heat and pain responses to palpation [S = 1 (1–1)]. The lameness score showed a normal gait [(S = 0 (0–0)] in 17 donkeys while 6 animals displayed intermittent, difficult-to-detect lameness [S = 1 (1–1)]. Following treatment, all animals (*n* = 23) in the three treatment groups achieved full clinical normalization of these specific parameters [S = 0 (0–0)] by T7, with these values remaining stable within the normal range throughout the rest of the study period.

**Fig. 1:**
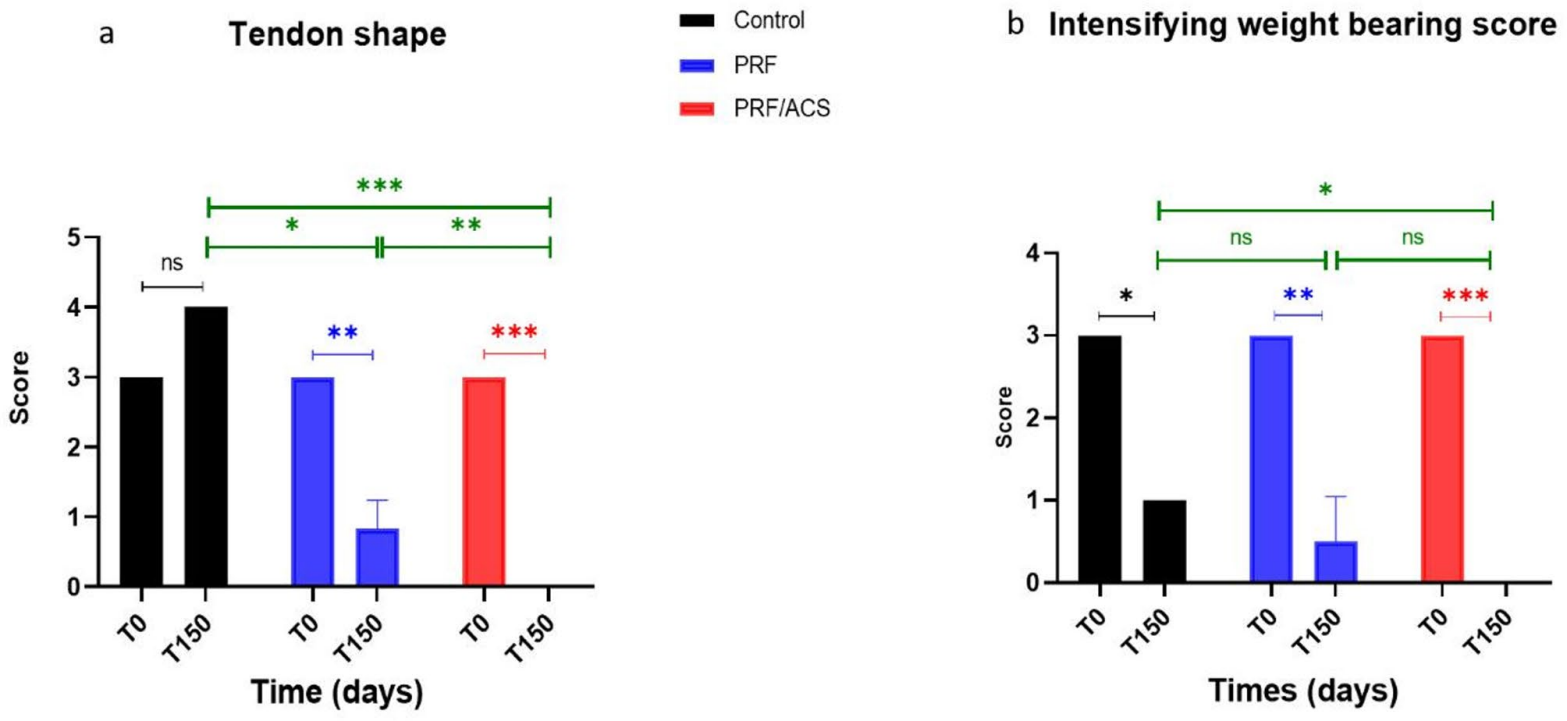
Showing the clinical evaluation of palpable shape of the tendons (**a**), and intensifying weight carrying score (**b**) in both control and treated groups at T0 and T150

Regarding tendon shape (Fig. 1a), all donkeys enrolled in the study exhibited an irregular tendon shape upon palpation at T0 [S = 3 (3–3). By the end of the study (T150), donkeys in the control group developed firm, nodular areas within the SDFT [S = 4 (4–4)], with a non-significant improvement from their T0 values (*P* = 0.083), yet a significant deviation from their normal tendon morphology (*P* = 0.014). In contrast, the PRF group demonstrated a marked improvement, with a reduction in score to [S = 1 (0–1)], which was statistically significant compared to both T0 (*P* = 0.020) and the control group (*P* = 0.046). The PRF/ACS group demonstrated the most substantial improvement at T150, with near-complete restoration of normal tendon shape [S = 0 (0–0)]. This outcome was significantly improved compared to T0 (*P* = 0.014), the control group (*P* < 0.0001), and the PRF group (*P* = 0.046), and was not statistically different from normal tendons (*P* = 1.000).

At T0, all animals exhibited notable limb offloading during the intensifying weight-carrying test [score = 3 (3–3)]. By T150, the control group showed a partial improvement, characterized by a mild preferential weight shift toward the contralateral limb [S = 1 (1–1); *P* = 0.014 vs. T0], yet still significantly different from normal limb loading (*P* = 0.014). In contrast, donkeys treated with PRF or PRF/ACS achieved near-complete restoration of weight distribution in response to axial loading [S = 0 (0–1) and 0 (0–0), respectively] at T150, with significant improvements compared to T0 (*P* = 0.024 and *P* = 0.014, respectively). Compared to the control group at T150, both PRF and PRF/ACS groups showed superior outcomes (*P* = 0.08 and *P* < 0.0001, respectively) in intensifying weight-carrying score. While the PRF group showed a non-significant difference from normal values (*P* = 0.083) at T150, the PRF/ACS group achieved complete normalization with no statistical difference from normal scores (Fig. 1b).

### Ultrasonographic findings

**Fig. 2:**
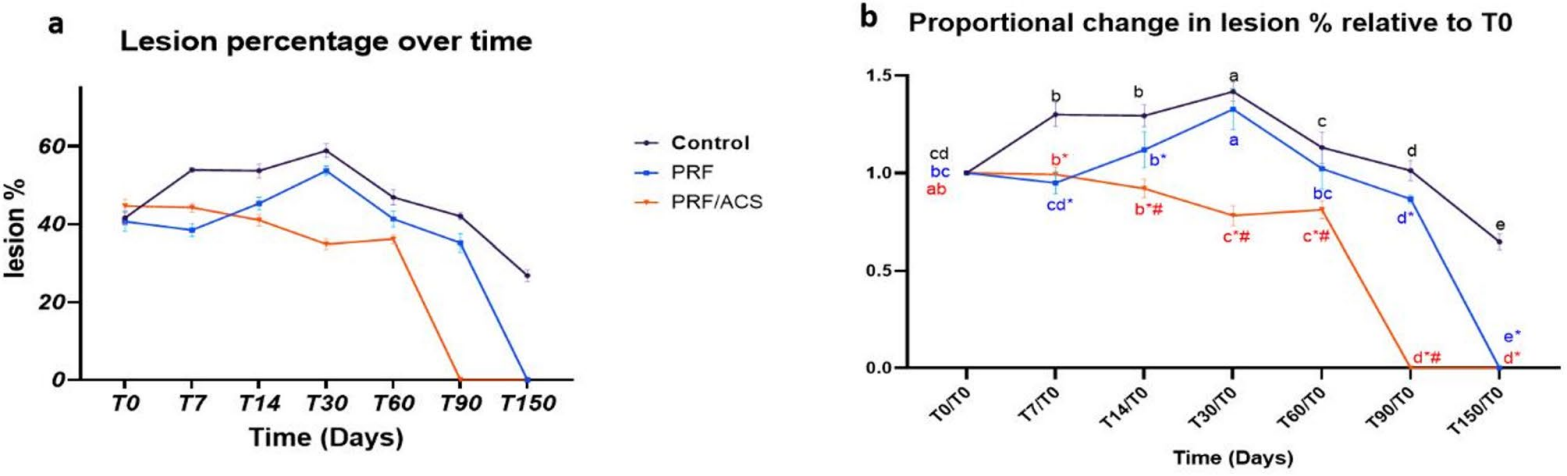
Showing the Lesion % (**a**) and proportional change in lesion % relative to T0 (**b**) in both control and treated groups. Times with different small letters are significant in the same group at *P* > 0.05. * There is a significant difference compared to the control group in the same time at *p* < 0.05. # There is a significant difference compared to the PRF group in the same time at *p* < 0.05

On the day of admission (T0), the mean T-CSA was 48.5 ± 1.3 mm² in the control group, 37.9 ± 1.2 mm² in the PRF group, and 49 ± 0.7 mm² in the PRF/ACS group. Correspondingly, the lesion cross-sectional area (L-CSA) was 20.2 ± 1.3 mm² in the control group, 15.4 ± 0.9 mm² in the PRF group, and 21.9 ± 0.8 mm² in the PRF/ACS group. The calculated lesion percentage (Fig. 2a) was 41.6% ± 1.8 in the control group, 40.6% ± 2.5 in the PRF group, and 44.7% ± 1.7 in the PRF/ACS group. Additionally, the contralateral limb’s T-CSA was 30 mm² in the control group, 27 mm² in the PRF group, and 29 mm² in the PRF/ACS group.

At T7 and T14, the proportional lesion percentage (PL%, Table 3; Fig. 2b) increased significantly in the control group compared to T0 (*P* = 0.0012 and *P* = 0.0009, respectively). In contrast, both PRF and PRF/ACS groups showed a non-significant change in the PL% at both T7 (*P* = 0.5790 and *P* > 0.9999, respectively) and T14 (*P* = 0.2776 and 0.1112, respectively) compared to T0. Intergroup comparisons of the PL% showed that both PRF and PRF/ACS group had significantly lower value than the control group at both T7 (*P* < 0.0001 for both groups) and T14 (*P* = 0.017 and *P* < 0.0001, respectively). However, PRF/ACS group showed a significant decrease in PL% at T14 (*P* = 0.0082) compared to the PRF group.

**Table 3:** Proportional changes in lesion percentage (PL%) overtime relative to T0 across the treatment groups

Evaluation times	Group		
	Control	PRF	PRF/ACS
T0/T0	1^cd^	1 ^bc^	1 ^ab^
T7/T0	1.3 ± 0.03 ^b^	0.95 ± 0.02 ^cd*^	0.99 ± 0.016 ^a*^
T14/T0	1.3 ± 0.02 ^b^	1.12 ± 0.04 ^b*^	0.92 ± 0.019 ^b*#^
T30/T0	1.4 ± 0.02 ^a^	1.31 ± 0.04 ^a^	0.78 ± 0.02 ^c*#^
T60/T0	1.1 ± 0.03 ^c^	1.02 ± 0.04 ^bc^	0.81 ± 0.019 ^c*#^
T90/T0	1.0 ± 0.02 ^d^	0.87 ± 0.01 ^d*^	0 ^d*#^
T150/T0	0.65 ± 0.02 ^e^	0 ^e*^	0 ^d*^

Times with different superscript letters ( a, b, c, d, e ) are significantly different at *p* < 0.05

^*^ there is a significant difference compared to the control group in the same time at *p* < 0.05

^#^ there is a significant difference compared to the PRF group in the same time at *p* < 0.05

At T30, PL% significantly increased in both control and PRF groups compared to T0 (*P* < 0.000 and 0.010, respectively), T7 (*P* < 0.0001 and *P* = 0.0101, respectively), and compared to T14 (*P* = 0.0309 and 0.0009, respectively), peaking during this time with no significant difference between them (*P* = 0.3115). In contrast, PRF/ACS group showed a significant lower PL% compared to both the control (*P* < 0.0001) and PRF (*P* < 0.0001) groups at T30. Additionally the PRF/ACS group demonstrated a significant reduction in P.L % at T30 compared to T0 (*P* = 0.0022).

At T60 onward, both control and PRF groups showed a gradual decline in lesion percentage at different rates. They exhibited a significant (*P* = 0.0029 and 0.0004, respectively) decrease in PL% at T60 compared to T30, while a non-significant difference (*P* = 0.7993) was observed in the PRF/ACS group at T60 compared to T30. The PRF group showed a first significant decrease in PL% below T0 by T90 (*P* = 0.0003), with a marked statistically significant difference compared to the control group (*P* < 0.0001) at T90, while the control group exhibited its first significant reduction at T150 (*P* < 0.0001) compared to T0.

The timing of lesion disappearance varied among treatment groups. In the control group, the anechoic core lesions resolved by T150 and replaced by hyperechoic dots. In contrast, complete lesion resolution in the PRF group occurred at T150. Notably, the PRF/ACS group exhibited the fastest recovery, with complete lesion disappearance observed as early as T90.

Concerning the proportional tendon cross sectional area (P.T-CSA, Table 4) at T14, all groups exhibited a significant increase compared to T0 (*P* = 0.0023 in control, 0.0077 in PRF, and < 0.0001 in PRF/ACS group). Thereafter, P.T-CSA values fluctuated, alternating between increases and decreases till the end of the study. By the end of the study (T150), both the PRF/ACS and control groups exhibited a significant reduction in P.T-CSA compared to T0 (*P* < 0.0001 for both groups). Otherwise, the PRF group showed a non-significant difference in P.T-CSA at T150 (*P* = 0.959) compared to T0.

**Table 4:** Showing statistical analysis of the proportional change in tendon cross sectional area (P.T-CSA) overtime relative to T0 across the treatment groups

Evaluation times	Group		
	Control	PRF	PRF/ACS
T0/T0	1.00 ^b^	1.00 ^c^	1.00 ^e^
T7/T0	1.05 ± 0.01 ^b^	1.12 ± 0.02 ^b*^	1.17 ± 0.01 ^bc*^
T14/T0	1.17 ± 0.02 ^a^	1.13 ± 0.02 ^b^	1.14 ± 0.01 ^cd^
T30/T0	0.96 ± 0.01 ^b^	1.24 ± 0.02 ^a*^	1.12 ± 0.01 ^d*#^
T60/T0	0.66 ± 0.01 ^d^	1.04 ± 0.02 ^c*^	1.19 ± 0.01 ^b*#^
T90/T0	0.92 ± 0.01 ^b^	0.80 ± 0.02 ^d*^	1.33 ± 0.01 ^a*#^
T150/T0	0.73 ± 0.01 ^c^	0.99 ± 0.006 ^c*^	0.69 ± 0.001 ^f #^

Times with different superscript letters ( a, b, c, d, e,f ) are significantly different at *p* < 0.05

^*^ there is a significant difference compared to the control group in the same time at *p* < 0.05

^#^ there is a significant difference compared to the PRF group in the same time at *p* < 0.05

**Fig. 3:**
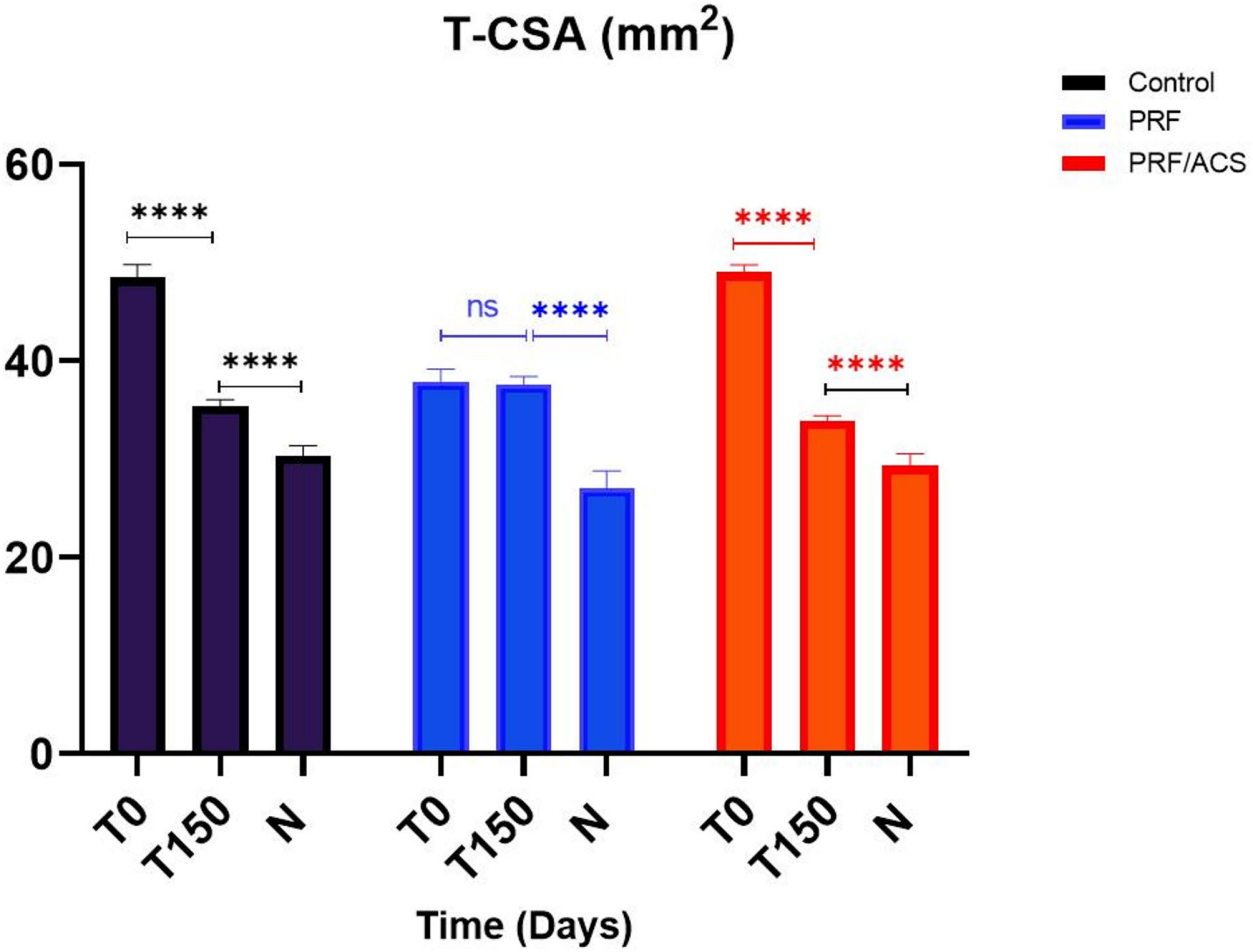
Showing T-CSA (mm^2^) of the affected limb in both control and treated groups at T150 compared to T0 using paired student t-test and compared to that’s of the contralateral limb (N) using unpaired student t-test

Comparing to the contralateral limb SDFT size (Fig. 3), all groups at the end of the study (T150) exhibited a significant (*P* < 0.0001 for all groups) increase in T-CSA. The PRF/ACS and control groups showed moderate increases of 15% (*P* < 0.0001) and 16% (*P* < 0.0001), respectively, while the PRF group exhibited a more substantial increase of 40% (*P* < 0.0001) relative to the contralateral limb.

**Fig. 4:**
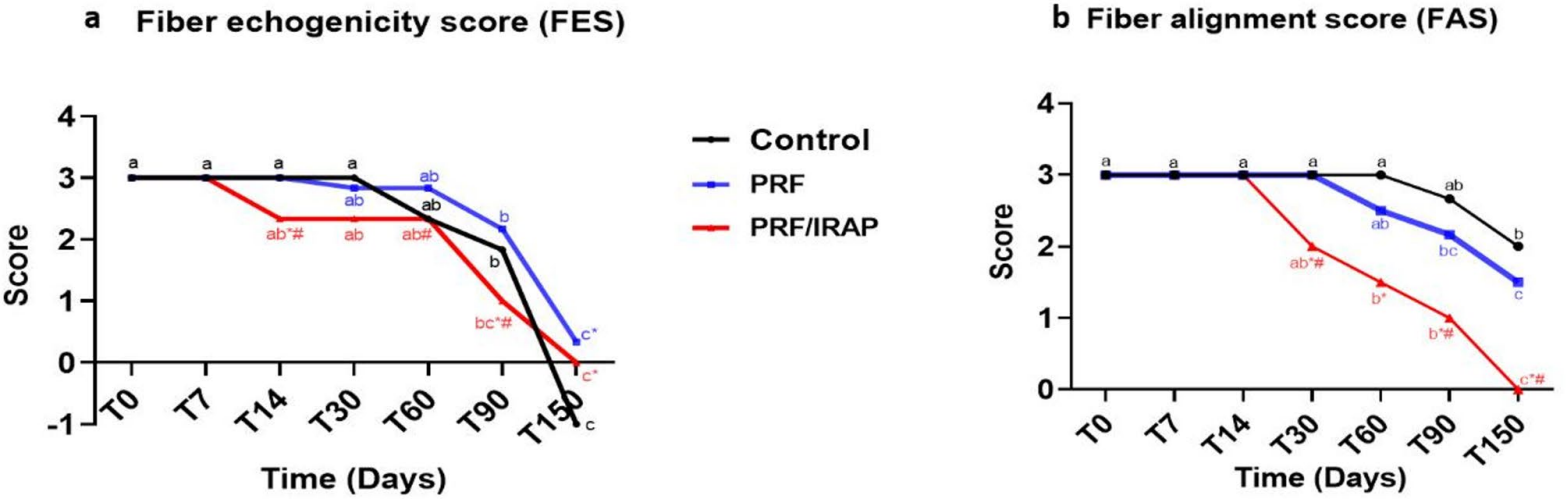
Showing the FES (**a**), and FAS (**b**) in both control and treated groups. Times with different small letters are significant in the same group at *P* > 0.05 using Friedman test. * There is a significant difference compared to the control group in the same time at *p* < 0.05. # There is a significant difference compared to the PRF group in the same time at *p* < 0.05 using Kruskal-wallis test

Compared to T0, FES (Fig. 4a) showed a significant decrease for the first time at T90 in all groups. At this time point, the PRF/ACS group exhibited significantly lower FES [S = 1 (1–1)] than both the control group [S = 2(1–2); *P* = 0.005] and the PRF group [S = 2(2–2); *P* = 0.001]. By T150, nearly normal fiber echogenicity was observed in both the PRF group [S = 0 (0–1)] and the PRF/ACS group [S = 0 (0–0)], with no significant difference between them (*P* = 0.393). In contrast, by the end of the study, the lesion area in the control group was replaced by hyperechoic dots, resulting in a significantly higher echogenicity compared to the PRF (*P* < 0.0001) and PRF/ACS (*P* = 0.003) groups.

**Fig. 5:**
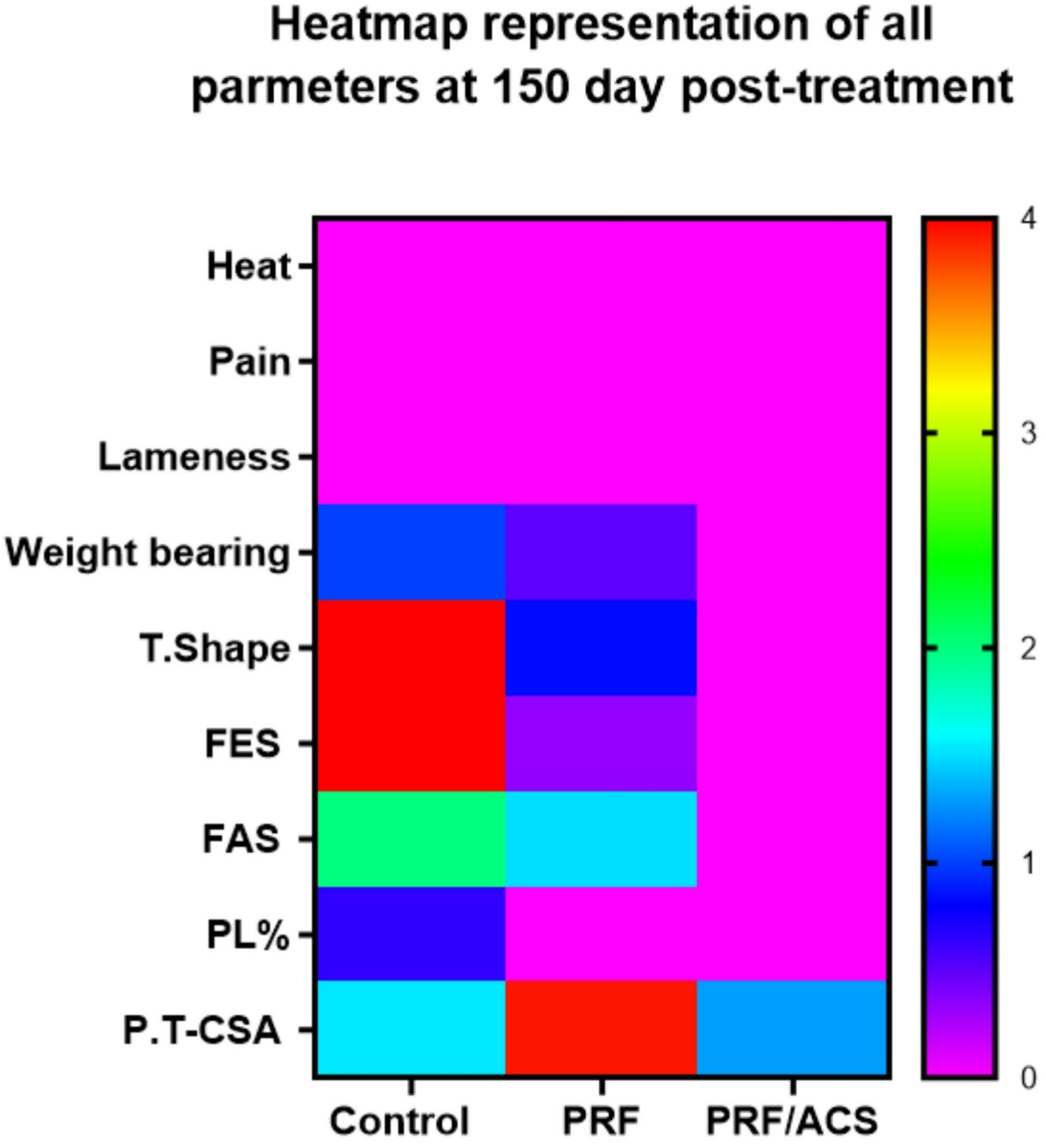
Heatmap reppresentation of all clinical and ultrasongraphical evaluation parameters in all groups at the end of the study (T150)

There was no significant difference in FAS (Fig. 4b) between the control and PRF groups at all time points (*P* > 0.05). In contrast, the PRF/ACS group demonstrated a significant decrease in FAS compared to the control group at T30, T60, T90, and T150 (*P* < 0.0001 for all time points), and compared to the PRF group at T30 (*P* < 0.0001), T60 (*P* = 0.026), T90 (*P* = 0.003), and T150 (*P* = 0.002). Compared to T0, the FAS in the control group significantly decreased for the first time at T150 (*P* = 0.004), representing fibers with less than 50% of the normal alignment pattern [S = 2(2–2)]. This parameter decreased significantly in the PRF group earlier at T90 (*P* = 0.02 vs. T0), achieving more than 50% fiber alignment pattern by T150 [S = 1.5(1–2)]. Notably, the PRF/ACS group exhibited an earlier and greater improvement, with a significant decrease in FAS at T60 (*P* = 0.045 vs. T0), achieving a nearly normal fiber alignment pattern [S = 0 (0–1)] by T150, and showing significantly better scores compared to both the PRF (*P* = 0.002) and control groups (*P* < 0.001) at T150 (Fig. 5).

## Discussion

This study aimed to evaluate the clinical efficacy of early administration of PRF alone or in combination with ACS in donkeys suffering SDF tendonitis. Donkeys were selected for this study due to the high local prevalence of SDF tendonitis cases. Such injuries closely resemble those observed in horses in terms of clinical signs, lesion characteristics, and prolonged healing [31]. Although the SDFTs in horses and donkeys share a high degree of anatomical and functional similarity, species- and use-related adaptations have been reported, particularly at the biochemical and mechanical levels. Moreover, the two species differ in tendon size and in the expression of pain-related behaviors [32–34]. Therefore, despite interspecies differences, the overall similarity in SDFT structure and function justifies the cautious extrapolation of the present findings from donkeys to horses [35, 36].

Results of this clinical trial highlight the superior effects of intralesional injection of I-PRF and ACS in promoting lesion resolution, enhancing fiber echogenicity and organization, and improving tendon shape and weight carrying capacity compared to the control group. The addition of ACS to I-PRF in the combination group led to faster lesion resolution (T90), and a significant enhancement in fiber alignment and T-CSA at T150 compared to PRF alone. I-PRF resulted in a significant reduction in lesion size and improvement in fiber echogenicity scores compared to the control group. In contrast, the control group exhibited the inferior healing, with hyperechoic dots formation at the end of the study suggesting fibrotic change. While T-CSA persistently increased in all groups relative to the contralateral limb, the PRF/ACS and control groups showed the most notable reduction by the end of the study (T150). In contrast, the PRF group maintained persistently elevated T-CSA levels, suggesting an ongoing remodeling process of tendon healing.

In this study, tendon healing was clinically evaluated using semi-quantitative scoring methods. While useful, these methods may lack absolute accuracy, which could be improved by integrating advanced techniques such as computerized gait analysis and thermography [37, 38]. These methods would provide a more objective and detailed evaluation of inflammation severity and tendon recovery. Although ultrasound is an effective and reliable tool for detecting and monitoring tendon injuries, as well as providing insights into lesion size, location, and extent [39], its accuracy depends on examiner expertise, controlled palpation pressure, and averaging multiple measurements to reduce variability. Unless these factors are carefully controlled, ultrasound may underestimate lesion dimensions and carry a higher risk of measurement error. Advanced imaging techniques such as MRI and CT can enhance precision by offering more detailed and objective assessments of tendon healing [40].

The low baseline (T0) scores for clinical evaluation parameters could be attributed to the diminution of acute inflammatory signs, as most animals were admitted 8.2 ± 2.8 days post-tendonitis onset. Consequently, no differences in these parameters were observed between groups throughout the evaluation period. These findings align with studies on naturally occurring and experimentally induced SDF tendonitis in donkeys and horses [36, 41]. However, other studies have reported different results, noting prolonged mild increases in these parameters post-treatment [25, 28, 42, 43].

The lack of significant differences in lameness scores among treatment groups does not indicate similar functional tendon repair, as these assessments that conducted during routine locomotion may not fully reflect the biomechanical resilience of the healed tendons. Since the SDFT experiences high loads during heavy drafting, structural differences may become more apparent later in rehabilitation as mechanical demands increase [44]. This notion is further supported by the intensifying weight-carrying results at the end of the study, which revealed significant improvements in biomaterial-treated groups, achieving even weight bearing distribution and indicating superior functional recovery compared to the control group. However, Alternative measurements such as acoustic radiation force impulse elastography [45] or direct tendon tensile strength measurements [46], may provide a more precise assessment of tendon biomechanical function. An intensifying weight-carrying method was developed in this study to evaluate the functional recovery of the SDFT in working donkeys under static load. It was inspired by frequent clinical observations in such cases where performance deficits appeared during load-bearing tasks (e.g., pulling heavily loaded carts or ascending inclines), despite the absence of visible lameness during regular walking. This approach was further supported by existing literature on working donkeys [47, 48], which reported that carrying mounted loads equivalent to 30% of their body weight (on average) can exert sufficient mechanical stress to reveal the functional capacity of the tendon.

The results of ultrasonographic assessment of absolute lesion size (mm^2^) represents a notable concern and is considered an unreliable indicator of tendon recovery due to pre-existing significant variation in tendon and lesion size among groups before treatment. Additionally, the resulted dynamic changes in tendon size during the healing process further complicate interpretations as discussed previously [49, 50]. These studies highlighted that increases in lesion size may be misinterpreted as pathological progression if the overall tendon has increased. To overcome these challenges, lesion percentage was estimated, as supported by several related studies [25, 51]. It considers individual variability in tendon and lesion sizes, provides a more reliable and accurate measure and reflects the extent of tendon healing over time. Significant differences in lesion percentages between groups on the admission day complicated statistical intergroup comparisons. Statistical standardization approach was applied allowing precise longitudinal evaluation of healing dynamics and identifies reliable significant differences in healing rates among treatment groups [28].

The significant increase in lesion percentage and T-CSA in the control group during the early weeks post-treatment reflects the natural inflammatory response to tendonitis. This process is driven by pro-inflammatory cytokines, particularly IL-1, which upregulates matrix metalloproteinases (MMPs) [52, 53] and other cytokines such as TNF-α and IL-6 [54, 55], which contribute to matrix degradation and lesion expansion. In contrast, the PRF group initially exhibited a minimal increase in lesion percentage and T-CSA during the first two weeks with a significant decrease compared to the control group, suggesting early stabilization of the injury site. This effect is attributed to PRF’s role in shifting macrophage polarization from a pro-inflammatory M1 phenotype to a pro-resolving M2 phenotype, leading to a reduction in IL-1β and IL-6 levels [56, 57]. Despite this early advantage, a significant increase in lesion percentage was observed at the T30 after treatment. This may be attributed to the transient nature of PRF’s anti-inflammatory effect, as its cytokines release is largely extending to 14 days [10]. This limitation suggests that PRF alone is insufficient to sustain long-term inflammatory regulation, resulting in trends similar to those seen in the control group. Among all treatment groups, the PRF/ACS group demonstrated the most favorable healing dynamics. A mild reduction in lesion percentage was noted within the first two weeks, which attributed to the PRF macrophage shifting and ACS’s targeted inhibition of IL-1 [21, 22, 24], facilitating a faster transition from the inflammatory to the reparative phase [19]. This positive trend continued with a significant decrease in lesion percentage at T30, indicating effective ECM turnover and regulation of inflammation [58].

The timing and extent of lesion percentage decline varied significantly between groups, highlighting the differential efficacy of the treatments. The PRF/ACS group exhibited the fastest lesion % decline, with a significant reduction observed by T30 and complete resolution as early as the T90 following treatment. This was accompanied by early improvement in fiber alignment and echogenicity scores by T90 after treatment, with the PRF/ACS group achieved a nearly normal fiber alignment pattern by the T150, significantly superior compared to the PRF group. This outcome reflects the synergistic effects of ACS’s targeted interleukin 1 inhibition [22] and PRF’s regenerative potential [59], which together optimize the repair environment and accelerate the healing process.

The PRF group showed delayed improvements, with a significant lesion % reduction occurring by T90 and complete resolution by T150. While echogenicity scores returned to normal pattern by T150, fiber alignment pattern remained less organized [S = 1.5 (1–2)]. This may be attributed to the transient nature of PRF’s anti-inflammatory and regenerative effect, as its cytokines release is largely extending to 14 days [10] and suggests that PRF alone is insufficient to fully regulate early inflammation, reduced its effectiveness compared to the PRF/ACS group. Additionally, this finding highlights the potential need for repeated PRF injections to maintain therapeutic efficacy.

In contrast, the control group exhibited a persistent increase in lesion percentage throughout most of the study period, with only minimal reduction observed toward the end (T150), where the lesion was replaced by hyperechoic dots with a misaligned fiber pattern (FAS = 2). Additionally, by T150, palpation revealed greater alterations in tendon shape in the control group (hard nodular area) compared to the treated groups, suggesting potential fibrotic changes. This may attributed to the persistent inflammatory condition and subsequent over-proliferation during the early repair phase which disrupted collagen synthesis, favoring weaker type III collagen over type I, ultimately leading to disorganized scar tissue and fibrosis [60–63], highlighting the tendon’s limited self-repair capacity without therapeutic strategies to regulate inflammation and enhance healing.

Biomaterial-treated groups exhibited significant improvements in tendon shape compared to the control group at the end of the study, indicating enhanced functional recovery. The absence of nodular fibrotic areas in the PRF and PRF/ACS groups suggests that these treatments promoted tendon remodeling by reducing fibrosis. Among the treatments, the PRF/ACS group demonstrated greater improvement in tendon shape than PRF alone, suggesting a potentially superior therapeutic effect.

Compared to contralateral SDFT size, the T-CSA in all groups was significantly higher throughout the observation period, a similar finding observed in other studies [28, 41, 51]. The fluctuating pattern of T-CSA observed in all groups reflect the dynamic interplay of tendon healing phases, where inflammation, proliferation, and remodeling overlap, resulting in fluctuating changes in T-CSA before a gradual decline toward the end of the study. Temporary rises indicated heightened cellular activity, conversely, declines indicated inflammation resolution and ECM remodeling [64]. However, by the end of the study (T150), the T-CSA exhibited varying degrees of reduction compared to T0 across all groups, further reflecting differences in treatment effects. The PRF/ACS and control groups showed significant decreases in this parameter, suggesting notable tendon remodeling but with notable differences in tissue quality observed ultrasonographically as discussed above. In contrast, the PRF group demonstrated a persistent increase in T-CSA with moderately aligned tendon fibers, suggesting ongoing extracellular matrix deposition and active tissue remodeling during healing, as previously attributed [40, 58, 65]. The PRF/ACS group showed a notable reduction in T-CSA and near-normal FAS at T150, indicating that the combination therapy may have promoted a more advanced remodeling stage and contributed to the reduction of tendon thickening. However, the similar reduction in T-CSA observed in the control group suggesting the involvement of other factors such as natural healing. Nonetheless, the clear differences in tissue quality observed ultrasonographically between the control and the combination groups, as discussed above, support the attribution of the T-CSA decrease to ACS.

## Conclusion

In donkeys with naturally occurring SDF tendonitis, intralesional injection of I-PRF combined with ACS led to earlier lesion resolution, better tendon shape, and improved FAS in comparison to both the saline (control) and I-PRF groups. These findings suggest a potential clinical advantage of the combination therapy in enhancing tendon healing, although further studies are needed to confirm its long-term efficacy.

## Study limitation

Histological evaluation is essential to confirm ultrasonographic and clinical findings. While ultrasound-guided tendon biopsy (minimal invasive technique) has been used in other equine studies [25], owner concerns about potential risks (tendon damage and reinjury) prevented its implementation in this study. A longer study duration is needed to fully assess rehabilitation outcomes and recurrence rates.

## Supplementary Information

Supplementary Material 1.

## Data Availability

No datasets were generated or analysed during the current study.

## Abbreviations

ACS: Autologous conditioned serum
IL-1Ra: Interleukin 1 receptor antagonist
IL-1: Interleukin 1
PRF: Platelet-rich fibrin
I-PRF: Injectable platelet-rich fibrin
SDFT: Superficial digital flexor tendon
SDF: Superficial digital flexor
S: Score
PL%: Proportional lesion percentage
P.T-CSA: Proportional tendon cross sectional area
