# Supplementary material for: Clinical benefits of early-stage autologous conditioned serum and injectable platelet-rich fibrin on healing superficial digital flexor tendonitis in donkeys
Source: Ir Vet J. 2025 Jun 7;78:13. doi: 10.1186/s13620-025-00299-y (PMC12144708; doi:10.1186/s13620-025-00299-y)
Supplement: Supplementary file 1 — Supplementary Material 1. [file 13620_2025_299_MOESM1_ESM.docx]

| **Table S1.** Modified ultrasound scoring system for evaluation FES & FAS | | |
| --- | --- | --- |
| **Score** | **Fiber echogenicity score (FES)**  **(Transverse plane)** | **Fiber alignment score (FAS)**  **(Sagittal plane)** |
| **-1** | Hyperechoic |  |
| **0** | Normoechoic  Normal to nearly normal echogenicity | Fibers with >75 % Alignment  Normal to near normal pattern |
| **1** | Hypoechoic  25- 50% loss of normal echogenicity | Fibers with 50–74 % of normal pattern Alignment |
| **2** | Mixed echogenicity  50% anechoic and 50% normal echogenic | Fibers with 25–49% of normal pattern Alignment |
| **3** | Mostly to completely anechoic | Fibers with <25 % of normal pattern Alignment |

| **Table S2**. Showing assessments of Tendon shape score upon palpation and Intensifying weight bearing at (T0) and after treatment (T150). | | | | | |
| --- | --- | --- | --- | --- | --- |
| Tendon shape upon palpation | | | | | |
|  | Control | PRF | PRF/ACS | Test statistic | P value |
| T0 | 3 (3-3) ^a^ | 3 (3-3) ^a^ | 3 (3-3) ^a^ | 0.00 | 1.000 |
| T150 | 4 (4-4) ^a^ | 1 (0-1) ^b*^ | 0 (0-0) ^b*#^ | 19.806 | 0.000 |
| Test statistic | 12.00 | 11.565 | 12.00 |  |  |
| P value | 0.002 | 0.003 | 0.002 |  |  |
| Intensifying weight bearing Response (Static examination) | | | | | |
| T0 | 3 (3-3) ^a^ | 3 (3-3)^a^ | 3 (3-3) ^a^ | 0.000 | 1.000 |
| T150 | 1 (1-1) ^b^ | 0 (0-1)^b*^ | 0 (0-0) ^b*^ | 16.867 | 0.001 |
| Test statistic | 12.000 | 11.143 | 12.000 |  |  |
| P value | 0.002 | 0.004 | 0.002 |  |  |
| Times with different superscript letters in the same group are significantly different at p<0.05.  *there is a significant difference compared to the control group and # there is a significant difference compared to the PRF group in the same time at p<0.05. | | | | | |

| **Table S3.** Showing the ultrasonographic results of tendon cross section are (T-CSA), lesion cross sectional area (L-CSA), and lesion percentage (Lesion %) at the admission day (T0). | | | | |
| --- | --- | --- | --- | --- |
|  | T-CSA | | L-CSA | Lesion % |
|  | Contralateral limb | T0 | T0 | T0 |
| Control | 30.3 ±0.422 | 48.5±1.3 | 20.2±1.3 | 41.6± 1.8 |
| PRF | 27 ±0.730 | 37.9±1.2 | 15.4±0.9 | 40.6± 2.5 |
| PRF/ACS | 29.3 ±0.494 | 49 ±0.7 | 21.9±0.8 | 44.7± 1.7 |

| **Table S4.** Ultrasonographic results of lesion percentage after treatment over time. | | | |
| --- | --- | --- | --- |
| **Evaluation times** | **Group** | | |
|  | Control | PRF | PRF/ACS |
| T0 | 41.5± 1.8 | 40.6± 2.5 | 44.7± 1.7 |
| T7 | 53.9± 0.5 | 38.4± 1.6 | 44.3± 1.1 |
| T14 | 53.7± 1.8 | 45.3± 1.6 | 41.0± 1.5 |
| T30 | 58.9±1.8 | 53.7± 1.2 | 34.8± 1.4 |
| T60 | 46.9± 1.9 | 41.3± 1.9 | 36.2± 1.1 |
| T90 | 42± 0.9 | 35.2± 2.3 | 0 |
| T150 | 26.9± 1.5 | 0 | 0 |

| **Table S5.** Showing results of Tendon Cross sectional Area (T-CSA) after treatment. | | | |
| --- | --- | --- | --- |
| **Evaluation times** | **Group** | | |
|  | Control | PRF | PRF/ACS |
| T0 | 48.5±1.3 | 37.9±1.2 | 49±0.7 |
| T7 | 50.9 ±0.6 | 44.6± 0.9 | 57.4 ±0.6 |
| T14 | 56.5±0.8 | 42.9± 1.0 | 55.9±0.8 |
| T30 | 46.4±0.8 | 46.8±1.6 | 54.9±0.6 |
| T60 | 31.8±0.9 | 39.4±0.9 | 58.2±0.5 |
| T90 | 44.6±1.1 | 30.4±0.7 | 65±0.6 |
| T150 | 35.4±0.6 | 37.6±0.8 | 33.9±0.5 |

| **Table S6.** Showing results of ultrasonographic assessment of SDFT fiber echogenicity score (FES). | | | | | |
| --- | --- | --- | --- | --- | --- |
| **Evaluation Times** | **Group** | | | | |
|  | Control | PRF | PRF/ACS | Test Statistic | P value |
| T0 | 3(3-3)^a^ | 3(3-3)^a^ | 3(3-3)^a^ | 0.00 | 1.000 |
| T7 | 3(3-3)^a^ | 3(3-3)^a^ | 3(3-3)^a^ | 0.00 | 1.000 |
| T14 | 3(3-3)^a^ | 3(3-3)^a^ | 2(2-3)^ab*#^ | 13.800 | 0.003 |
| T30 | 3(3-3)^a^ | 3(2-3)^ab^ | 2(2-3)^ab^ | 7.667 | 0.053 |
| T60 | 2(2-3)^ab^ | 3(2-3)^ab^ | 2(2-2)^ab #^ | 10.062 | 0.018 |
| T90 | 2(1-2)^b^ | 2(2-2)^b^ | 1(1-1)^bc *#^ | 16.611 | 0.001 |
| T150 | -1(-1/-1)^c^ | 0(0-1)^c *^ | 0(0-0)^c *^ | 18.254 | 0.000 |
| Test Statistic | 41.425 | 40.384 | 43.215 |  | |
| P value | 0.000 | 0.000 | 0.000 |  |  |
| Times with different superscript letters in the same group are significantly different at p<0.05. df = 6 and test statistic of Freidman test.  *there is a significant difference compared to the control group and # there is a significant difference compared to the PRF group in the same time at p<0.05. df = 2 and test statistic of Kruskal-Wallis. | | | | | |

| **Table S7.** Showing results of ultrasonographic assessment of SDFT fiber alignment score (FAS). | | | | | |
| --- | --- | --- | --- | --- | --- |
| **Evaluation Times** | **Group** | | | Test Statistic | P value |
|  | Control | PRF | PRF/ACS |  |  |
| T0 | 3(3-3)**^a^** | 3(3-3) **^a^** | 3(3-3) **^a^** | 0.000 | 1.000 |
| T7 | 3(3-3)**^a^** | 3(3-3) **^a^** | 3(3-3) **^a^** | 0.000 | 1.000 |
| T14 | 3(3-3)**^a^** | 3(3-3) **^a^** | 3(3-3) **^a^** | 0.000 | 1.000 |
| T30 | 3(3-3)^a^ | 3(3-3) **^a^** | 2(2-3) **^ab*#^** | 23.000 | 0.000 |
| T60 | 3(3-3)**^a^** | 3(3-3) **^ab^** | 2(2-2) **^b*^** | 13.636 | 0.003 |
| T90 | 3(2-3)**^ab^** | 3(2-3) **^bc^** | 2(2-2) **^b^**^*#^ | 18.708 | 0.000 |
| T150 | 2(2-2)**^b^** | 1.5(1-2) **^c^** | 0(0-1) **^c^** ^*#^ | 19.499 | 0.000 |
| Test Statistic | 46.112 | 43.907 | 52.825 |  |  |
| P value | 0.000 | 0.000 | 0.000 |  |  |
| Times with different superscript letters in the same group are significantly different at p<0.05. df = 6 and test statistic of Freidman test.  *there is a significant difference compared to the control group and # there is a significant difference compared to the PRF group in the same time at p<0.05. df = 2 and test statistic of Kruskal-Wallis. | | | | | |
